# Mental and Oral Health: A Dual Frontier in Healthcare Integration and Prevention

**DOI:** 10.7759/cureus.76264

**Published:** 2024-12-23

**Authors:** Khairunnisa Z, Sibyl Siluvai, Keerthana Kanakavelan, Leema Agnes, Indumathi KP, Krishnaprakash G

**Affiliations:** 1 Public Health Dentistry, Sri Ramaswamy Memorial (SRM) Kattankulathur Dental College and Hospital, Sri Ramaswamy Memorial Institute of Science and Technology (SRMIST), Chennai, IND

**Keywords:** dental pain, depression, effects on general health, gum disease, mental health, oral health, preventive care

## Abstract

Mental and oral health are interrelated, and problems in one area usually affect the other. This review discusses the complex relationships between oral and mental health, particularly the psychosocial challenges faced by individuals with mental health disorders in maintaining oral hygiene, including stigma, lack of access to care, and financial barriers. It also discusses how psychiatric conditions influence oral health, with regard to issues such as dry mouth, gum disease, and tooth decay, and how poor oral health can aggravate mental well-being. Preventive measures, treatment approaches, and solutions to overcome these barriers are also discussed.

Methodological rigor was achieved by systematically searching high-impact journals indexed in PubMed and Google Scholar for peer-reviewed publications published between 2000 and 2024. High-impact journals have a high impact factor and rigorous peer-review standards. Articles that did not undergo peer review, articles published in languages other than English, or those that were not accessible (because access was closed unless key information was available through abstracts or summaries) were excluded. It mentions the relevance of integrating mental health services with oral health services, with a focus on individualized care, interdisciplinary cooperation, and creative strategies to break down systemic barriers, resulting in better health outcomes and lower healthcare costs.

## Introduction and background

Mental health was defined by the World Health Organization (WHO) as a state of well-being in which individuals realize their potential, work productively, deal with the daily stress of life, and contribute to their respective communities [1]. This concept highlights how the importance of mental health relates to overall well-being. The mental health disorders of anxiety, depression, and schizophrenia are frequently brought on by systemic inflammation, medication side effects, and lifestyle modifications. These conditions have a substantial impact on many facets of our health, including our oral and physical health [2]. The condition of the teeth, periodontium, and oral-facial system - often known as oral health [3] - influences masticatory function, aesthetic smile, and speech. Individuals experiencing severe mental health difficulties often struggle with poor oral health, which can exacerbate their mental health issues [4]. This is due to factors such as pain, infections, and neglect of oral hygiene. Studies indicate that people experiencing mental health challenges face a greater chance of developing conditions such as dental caries, gum disease, and edentulism (tooth loss) [5,6]. Oral and mental health have a linked and complicated relationship. People with severe mental disorders, like bipolar disorder and schizophrenia, have a higher chance of poor dental hygiene and tooth loss due to factors like impaired well-being, psychiatric treatment side effects, and persistent stress [4-6]. Moreover, changes in lifestyle brought about by mental health problems - such as inadequate diet, excessive carbohydrate consumption, and substance use - could aggravate oral health problems [3-5]. The findings of the research study conducted by Matevosyan (2010) support an integrated treatment that includes both mental health and oral health dimensions, highlighting that individuals with severe mental disorders often experience significantly poor oral health outcomes due to drug side effects, neglect of hygiene practices, and limited access to dental care [6]. In this context, Kisely (2016) advanced the idea of suggesting, "There is no mental health without oral health," which further consolidates the concept among professionals about the interdependency of both disciplines [7]. It has been observed that the application of an integrated approach of this nature, which takes into account treatment based on both mental health and dental support, has aided in improving the overall well-being of patients afflicted with the former. A relevant example of an integrated care model is co-location, where mental health professionals work alongside dental care providers in shared facilities. Co-location promotes good interprofessional communication and enables the development of proper care plans to meet each patient's individual needs [3]. This co-located care and training program for dental professionals concerning the effects of psychiatric disorders and their pharmacological treatments has evidenced significant improvement in the quality of care rendered to those with mental health issues [3]. Furthermore, the integration of oral health education into mental health services may reduce barriers to care, since mental health and oral health service use have a strong association with one another [2]. These include salivary stimulants and fluoride therapies, which are effective in reducing the xerostomic effects caused by psychiatric medications [5]. Community health centers providing dental and mental health services are pivotal in ensuring that individuals who suffer from severe mental illnesses receive regular oral health assessments [6]. A well-informed dentist will be able to implement preventive measures, such as fluoride varnishing and tailored oral hygiene counseling [5]. According to Hansen et al. (2021) [4], integrated care strategies focus on systemic barriers but, in the meantime, have improved access and outcomes for vulnerable populations. Such models provide patient-centered care that realizes the full potential of health. Research shows that dental health issues, such as periodontitis, can promote systemic inflammation, worsening mental health issues like anxiety and depression. Similarly, untreated mental health conditions can result in neglect of dental health care, producing a feedback loop of declining health [2,6]. The correlation between oral and mental health would thus be examined for the possibility of early diagnosis of comorbid conditions, creating an opportunity for timely interventions. For instance, the treatment of chronic periodontal disease reduces systemic inflammation, which is often seen in depression and anxiety [5]. Similarly, attention to mental health care can significantly enhance adherence to dental practices, which further leads to better general health outcomes [4]. Gathering knowledge about mental and oral health promotes overall health improvement. Better dental health, for example, boosts self-esteem and reduces social disengagement, which is frequently associated with mental illnesses [8]. Proper mental health care helps bring about good oral health through regular dental hygiene practices. Similarly, public health programs could also make effective use of this information for prevention, reduction of healthcare costs, and improved outcomes for patients [3,7].

## Review

Aim of this review

This review aims to examine the complex relationships between mental and oral health. It specifically points out psychosocial challenges in the maintenance of oral hygiene for individuals suffering from mental health disorders, such as stigma, lack of access to care, and financial barriers [3,7]. This review further highlights the contribution of psychiatric treatments, particularly the side effects of some medications, such as xerostomia, periodontal disease, and caries, which contribute to exacerbating oral health conditions. This would thereby align preventive care strategies, which are part of integrated care models, with personalized treatment methods, as shown in the effective reduction of disparities between mental and oral health, leading to an improved connection to comprehensive well-being [6,7].

Search strategy

This narrative review involves a systematic search to investigate the complex relationship between mental and oral health. The references included in this review were searched and compiled using a comprehensive strategy, employing PubMed and Google Scholar. The search strategy involved using combinations of relevant keywords, such as “mental health,” “oral health,” “depression,” “dental pain,” and “preventive care.” Boolean operators, like AND, OR, and NOT, were applied to refine the search queries and ensure the inclusion of comprehensive and diverse studies.

Filters were applied to peer-reviewed articles, reviews, and systematic reviews published in high-impact journals between the last two decades (2000-2024), as well as open-access and relevant studies. High-impact journals were defined as those indexed in PubMed or Google Scholar with a high impact factor and highly regarded for their strict review process. Articles not peer-reviewed, published in languages other than English, or not accessible due to closed access - unless key findings could be ascertained from abstracts or summaries - were excluded. The study was mainly focused on research that had specifically addressed psychosocial barriers, the effects of psychiatric medications on oral health, and strategies for integrated care. This study involved qualitative and quantitative research, thus ensuring a comprehensive, multidisciplinary approach. PubMed provided clinical knowledge and healthcare systems, whereas Google Scholar offered multidisciplinary studies. Methods in the papers give a view of psychosocial, biological, and systemic factors that influence dental and mental health.

Impact of mental health conditions on oral health

Individuals with mental health conditions often suffer from depression, anxiety, and other severe mental illnesses, which pose serious challenges in their quest to maintain oral hygiene. This nexus is attributed to behavioral, biochemical, and socioeconomic factors, including diminished motivation for self-care, altered salivary flow due to medication, and barriers to accessing dental care [9,10].

Literature shows that poor oral health care exacerbates the conditions of mental health that drastically debilitate quality of life [11,12]. Patients suffering from psychiatric disorders exhibit low motivation, which results in poor hygiene and facilitates a rise in dental issues. Poor oral hygiene has been linked with depression and schizophrenia. These groups are more susceptible to dental caries and periodontal disease [9]. Behavioral factors also apply. The symptoms that might present, such as apathy and fatigue, and many other cognitive impairments associated with mental health conditions, will prevent the person from maintaining good oral hygiene. According to Patel and Gamboa (2012), "dental anxiety and difficulty in accessing dental care add to the problem" [13]. In addition, patients suffering from mental conditions often engage in unhealthy behaviors toward their oral health, such as poor dietary habits and substance abuse, which have significant effects on oral health outcomes [14]. Additionally, psychotropic drugs commonly used in the treatment of mental illness, such as antidepressants and antipsychotics, induce xerostomia, increasing the risk of dental caries. A decrease in salivary flow weakens the normal protective mechanisms of the oral cavity, thus augmenting the prevalence of caries and periodontal disease, which require enhanced dental care for these patients [11,15]. Moreover, bruxism, or teeth grinding, which results from antipsychotic medication, also contributes to erosion of enamel and temporomandibular joint disorders (TMJDs) [16].

Psychopharmacologic drugs and treatments for mental illnesses significantly impact oral health, affecting physiological processes as well as behavioral acts. Many psychotropics, including antidepressants and antipsychotics, create xerostomia, or dry mouth, which decreases the production of saliva and increases the risk of dental caries and periodontal disease. For patients on long-term psychotropic medication regimens, Ciancio (2004) urges preventive oral care to balance out these effects [17].

Furthermore, the most significant impact of anxiety or dental phobia is found in the irregularity or avoidance of routine dental visits, consequently worsening oral health problems [12]. Poor oral health has a negative effect on social well-being and self-esteem. Psychological distress is experienced by individuals with chronic oral health problems [10,12]. This parallelism brings to light the necessity of promoting the interlinking of mental health and oral health for the betterment of life quality in these individuals [18]. These conditions are linked to socioeconomic challenges, limited access to dental care, and reduced general health status in individuals with mental disorders [18,19].

Financial, stigma-related, and lack of integrated-care models are a few reasons why most people with mental health conditions avoid dental services. Integrated care models, which bring together dental and mental health services, have been shown to be effective in improving oral health outcomes, especially for vulnerable populations. One example is the co-location model, whereby medical and dental providers work in the same physical space. This model, suggested by Tiwari et al. (2021) [2], ensures seamless referrals and integrated care, resulting in better access to services and enhanced teamwork among healthcare professionals. It fosters holistic care, which is especially important for individuals with complex health needs, as it addresses both physical and mental health simultaneously [2]. McCay (2019) discussed an approach, which is embedding behavioral health consultants within dental practice settings. This model is designed to offer immediate mental health support to dental patients, which could range from addressing dental anxiety and depression to substance use within the dental setting. This approach is especially helpful for patients with chronic mental health or substance use issues, as it ensures they receive comprehensive care in one setting, thereby improving overall health outcomes. These integrated care models acknowledge the psychological factors that affect oral health, promoting better access to care and improved overall well-being for these vulnerable groups [1,2]. More importantly, oral health education, as well as tailored intervention strategies, like salivary stimulants and fluoride treatments, are crucial in offsetting the pathological effects of psychiatric medications on oral health [5].

Patients with schizophrenia are at high risk for dental diseases, with studies suggesting that their dental health may be better than the general population's. Periodontal disease in these patients can be linked to subclinical atherosclerosis, which may increase their risk of developing cardiovascular disease [20]. Other factors that have a bearing on the oral health of these patients include a low frequency of tooth brushing, fewer visits to the dentist, smoking, and poor nutrition [13].

Mental disorders have been related to tobacco and alcohol use, as well as poor diet and oral hygiene. People with mental disorders may use tobacco (nicotine) and alcohol for self-medication. Mental disorders, such as depression, have also been related to sugar cravings, eating dysregulation, and poor diet [8].

Few research studies have reported on the association between poor mental health and poor oral health status. However, a few studies in this area shed light on possible underlying reasons for this association. Low prioritization of oral health, low recognition of the association between poor oral and mental health by healthcare providers, and lack of alternative service models in dental settings for those with heightened fear, anxiety, and/or mental health problems have been identified as some of the underlying reasons for this association [2].

Impact of oral health on mental well-being

Research has shown that poor oral health, in the form of untreated caries and periodontitis, affects mental health negatively. Such issues cause social avoidance and low self-esteem. It has been noted by Shekarchizadeh et al. (2013) that patients suffering from chronic dental pain are more stressed and have more psychological symptoms, which can lead to depression [21]. Wadhawan et al. (2020) reported that patients diagnosed with chronic periodontitis had higher levels of anxiety and depression [22]. Untreated decay among adolescents was linked to lower self-esteem and increased psychological distress, and it is evident that dental conditions affect the mental well-being of adolescents - a factor Plessas et al. (2022) also pointed out in their discussion [23].

A significant number of studies have shown that a deep relationship exists between oral health and self-esteem. According to Reisine (1988), the aesthetics of the teeth are very important in determining self-image and social confidence [24]. Visible dental problems, like missing teeth or discoloration, cause discomfort reactions and social exclusion, thereby lowering esteem levels. According to Campos et al. (2020), obvious dental problems would make people abstain from visiting social gatherings for fear of others' opinions about them [25].

This is further supported by other research, whereby most of the patients who experience severe oral problems tend to have exaggerated social anxiety; hence, bad relationships are created among the affected individuals, according to Kalaigian and Chaffee (2023) [26].

Dental pain is thus an indicator of poor oral health and is related to psychological well-being. TMJD pain, along with untreated cavities, increases the symptoms of anxiety and depression. For patients, chronic dental pain is very distressing and typically follows the pattern of mood alteration, which affects the patient's ability to function. Hariyani et al. (2024) said that chronic dental pain elevates the perception of stress, and patients become more sensitive to mental illness [27].

Giannakopoulos et al. (2010) also indicated that depression exacerbates chronic dental pain, and vice versa. Preventive care, such as brushing, flossing, and regular dental visits, can support both oral and psychological health [28]. According to Hallett et al. (2023), good oral hygiene improves self-esteem and helps with mental health [29]. Ylöstalo et al. (2003) stated that proper oral hygiene enhances the life of patients, thereby increasing satisfaction and lowering the levels of distress among patients [30]. Proper dental care and hygiene maintain the quality of life, thereby preventing psychiatric disorders related to oral health issues, according to Plessas et al. (2022) [23].

Impact of psychiatric medications on oral health

One of the most frequent oral adverse effects of psychiatric drugs is xerostomia, commonly known as dry mouth. It encompasses several pharmacological classes. Selective Serotonin Reuptake Inhibitors (SSRIs) are among the most widely used drugs in the treatment of depression and anxiety and have been associated with some decline in salivary flow, which causes xerostomia. Consequently, this condition may raise the risk of dental caries, periodontal disease, and the establishment of other oral infections [31]. Similarly, Tricyclic Antidepressants (TCAs) and Serotonin-Norepinephrine Reuptake Inhibitors (SNRIs) have been associated with a decreased salivation condition that not only favors the deposition of dental plaque but may also cause other changes in the sense of taste and gingival enlargement [32]. Details of medications and their oral health implications are summarized in Table 1 [33-38].

**Table 1:** Role of medications and treatments for mental health on oral cavity Table credit: [33-38]

Medications	Side effects	Oral cavity
Selective Serotonin Reuptake Inhibitors (SSRIs) usage: depression, anxiety disorders	Nausea, headache, sexual dysfunction	Dry mouth, taste changes, gingival overgrowth
Tricyclic Antidepressants (TCAs) usage: depression, chronic pain, migraines	Drowsiness, constipation	Xerostomia, taste changes, gingival overgrowth, difficulty swallowing
Serotonin-Norepinephrine Reuptake Inhibitors (SNRIs) usage: depression, anxiety disorders, chronic pain	Dizziness, insomnia, increased heart rate	Xerostomia, taste changes, gingival overgrowth
Atypical Antipsychotics usage: schizophrenia, bipolar disorder	Weight gain, drowsiness, restlessness	Tardive dyskinesia, gingival overgrowth
Benzodiazepines usage: anxiety disorders, insomnia, seizure disorders, such as epilepsy	Drowsiness, impaired coordination, memory impairment	Xerostomia, plaque buildup, and tooth decay
Mood stabilizers (e.g., lithium) usage: bipolar disorder	Tremor, increased thirst, weight gain	Metallic taste, geographic tongue (smooth, red patches on the tongue) or black hairy tongue (a buildup of bacteria on the tongue), swelling of the salivary glands
Stimulants (e.g., methylphenidate) usage: attention-deficit/hyperactivity disorder (ADHD), narcolepsy	Insomnia, loss of appetite, increased heart rate	Mouth sores or ulcers, teeth grinding, difficulty swallowing
Antipsychotics (e.g., clozapine) usage: schizophrenia, bipolar disorder	Drowsiness, weight gain, seizures	Changes in taste, increased plaque buildup, tooth decay, and gum disease, bruxism, involuntary movements of the tongue, lips, and jaw, which can be disfiguring and painful.
Antiepileptics (e.g., phenytoin) usage: epilepsy, seizures	Coordination problems, vision problems, constipation, skin rash, hair growth changes	Gingival hyperplasia, dental caries
Antihistamines usage: allergies, insomnia	Headache, difficulty urinating, rapid heart rate, blurred vision	Sore throat, gum disease, tooth decay

Long-term usage of corticosteroids, such as methylprednisolone and prednisone, results in systemic effects on the bone, with osteoporosis being the most common effect. However, it also affects alveolar bone and oral health. Cockburn et al. (2017) and Abed et al. (2023) report that systemic drugs, including corticosteroids, can contribute to bone loss and periodontal complications [35,37]. Similar evidence has been found regarding patients who are ambulatory and taking enzyme-inducing antiepileptic drugs, such as phenytoin, phenobarbital, carbamazepine, and primidone, compared to those taking non-enzyme-inducing drugs, such as valproic acid, lamotrigine, clonazepam, gabapentin, and topiramate [17].

Treatment of mental disorders and their interaction with oral health requires cooperation between mental health and dental caregivers. Multidisciplinary approaches ensure comprehensive care, improving both mental and oral health outcomes for patients.

Barriers to oral health care for individuals with mental health conditions

Individuals with mental health conditions encounter numerous barriers when seeking oral health care. These barriers stem from stigma, financial constraints, lack of integration between health services, inadequate training among dental professionals, and systemic issues, such as geographic inaccessibility. The stigma associated with mental health conditions often prevents individuals from seeking care, while biases among dental providers further exacerbate the problem. Sensitivity training for dental professionals is essential to address these biases and ensure inclusive care environments [39].

Financial constraints significantly hinder access to dental care for individuals with mental health disorders, who are often unemployed or underemployed. Expanding insurance coverage and government subsidies could alleviate this challenge [40]. Additionally, there is a lack of integration between mental health and oral health services. This disconnect prevents a holistic approach to patient care, as mental health professionals and dental providers rarely collaborate. Integrated care pathways can help bridge this gap and improve outcomes.

The training deficits among dental professionals further compound the issue. Many practitioners lack the skills to effectively manage patients with mental health conditions, leading to unmet care needs. Curricular reforms that incorporate mental health modules into dental education can empower providers with the necessary knowledge and empathy [40]. Accessibility is another significant barrier, particularly in rural areas, where transportation challenges and limited clinic availability hinder care. Mobile dental units could provide solutions for these underserved regions [5]. Psychological factors also play a crucial role. Dental anxiety and phobia are common among individuals with mental health conditions, creating additional obstacles to seeking care. Trauma-informed care practices could help address these concerns and encourage patients to engage with dental services [41].

Though the association between oral health and mental health is bidirectional, both of them considerably affect each other in terms of their shared risk factors. Depressive and anxiety-related mental health disorders are often correlated with a decline in initiative and oral hygiene practices for the maintenance of health, which consequently increases the incidence of both caries and periodontitis. Psychiatric medications contribute to xerostomia, thereby exacerbating conditions related to oral health. Poor oral health has been linked to low self-esteem and impaired social interaction, thus increasing psychological distress. Higher levels of depression and anxiety have been reported due to chronic pain in the mouth. The studies suggest that there is a need for an integrated oral and mental health care delivery system, with a preventive approach through fluoride application and specially designed patient education. Also, targeted interventions and institutional reforms could lessen the aggregate effect of these health problems and be welfare-enhancing [2,42].

**Figure 1:**
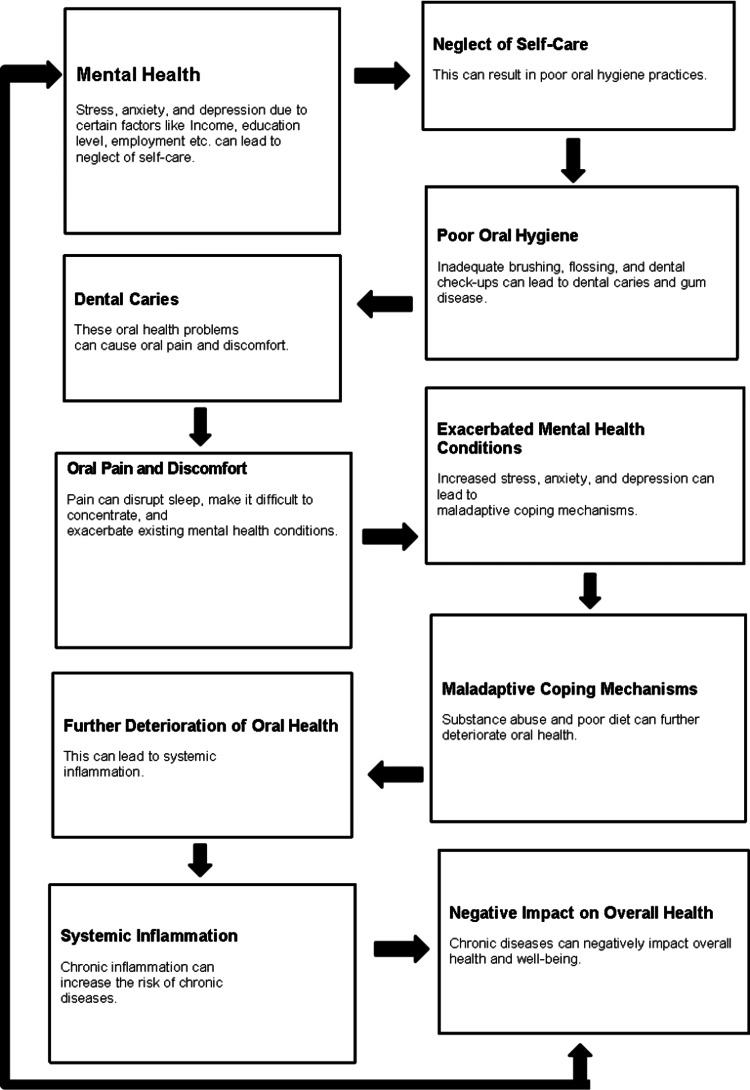
Pathways linking mental health and oral health [4] Image credit: Leema Agnes

The relationship between dental health and mental health is interdependent. As shown in Figure 1, factors like periodontitis and caries can arise from inadequate mental health, which leads individuals to neglect self-care practices, such as maintaining proper oral hygiene. Some experts note that the impact of this is significant: mental illness can be exacerbated by discomfort and pain in the mouth. All the ill-health conditions, scattered throughout a period, create vicious cycles that ultimately affect an individual's general health and well-being in profound ways [4].

Addressing these barriers requires systemic changes, including increased funding, integrated care models, targeted education, and outreach initiatives such as telehealth services and mobile clinics. By tackling these issues comprehensively, oral health outcomes for individuals with mental health conditions can be significantly improved.

## Conclusions

This interaction between mental health and oral health is very complex, thereby making a holistic approach to healthcare very important. Mental health disorders, such as schizophrenia, depression, and anxiety, impair oral health through decreased self-care, side effects of medication (such as xerostomia), and behavioral problems, which result in conditions such as dental caries, periodontitis, and tooth loss. Policymakers and clinicians need to have strategies on how to approach the issue. Mental health and dental practitioners working together could produce integrated care models targeting the needs of the patients. Co-located services or behavioral health professionals in dental clinics deliver full-scale care. These approaches enhance comprehensive care and access, with an emphasis on prevention and individualization. Patient education should include mental and oral health treatments. Incorporating mental health modules into dental training could reduce stigma and prepare practitioners for empathetic practice. Mobile dental units, telehealth, and insurance can help overcome financial and geographic barriers that otherwise limit access to such a group of patients, thus drastically improving mental and oral health in co-occurring patients. The issues of how digital solutions can fill the gap in care, and how culture and socio-economic factors impact access to treatment, will inform policies regarding health equity and reducing disparities. This conclusion underlines the importance of action, while also emphasizing areas for future research and structural improvements.
